# N-Terminal and C-Terminal Domains of Calmodulin Mediate FADD and TRADD Interaction

**DOI:** 10.1371/journal.pone.0116251

**Published:** 2015-02-02

**Authors:** Giuliana Papoff, Nadia Trivieri, Sonia Marsilio, Roberta Crielesi, Cristiana Lalli, Loriana Castellani, Edward M. Balog, Giovina Ruberti

**Affiliations:** 1 National Research Council, Institute of Cell Biology and Neurobiology, Campus Adriano Buzzati-Traverso, Monterotondo, Rome, Italy; 2 Department of Human Sciences, Society and Health, University of Cassino, Cassino, Italy; 3 School of Applied Physiology, Georgia Institute of Technology, Atlanta, Georgia, United States of America; University of Alabama at Birmingham, UNITED STATES

## Abstract

FADD (Fas–associated death domain) and TRADD (Tumor Necrosis Factor Receptor 1-associated death domain) proteins are important regulators of cell fate in mammalian cells. They are both involved in death receptors mediated signaling pathways and have been linked to the Toll-like receptor family and innate immunity. Here we identify and characterize by database search analysis, mutagenesis and calmodulin (CaM) pull-down assays a calcium-dependent CaM binding site in the α-helices 1–2 of TRADD death domain. We also show that oxidation of CaM methionines drastically reduces CaM affinity for FADD and TRADD suggesting that oxidation might regulate CaM-FADD and CaM-TRADD interactions. Finally, using Met-to-Leu CaM mutants and binding assays we show that both the N- and C-terminal domains of CaM are important for binding.

## Introduction

Signal transduction pathways controlling immunity, inflammation and apoptotic or necroptotic cell death depend to a large extent on proteins containing homotypic interaction domains belonging to the death-fold superfamily [1, 2]. This superfamily consists of receptor, adaptor, effector and inhibitor proteins containing protein-protein interaction modules: death domain (DD), death effector domain (DED), caspase recruitment domain (CARD) and pyrin domain (PYD) that characterize four subfamilies. Hallmark of the superfamily is a protein-protein interaction domain structure, the so-called death-fold, which consists of a globular structure wherein six amphipathic α-helices are arranged in an antiparallel α-helical bundle with Greek key topology [3–6]. Variations in length and orientation of the α-helices as well as distribution of charged and hydrophobic residues at the surface are small among members of each subfamily. Death-fold domains are involved in the assembly of multimeric complexes leading to activation of key effectors such as caspases and kinases [1, 2].

Members of the death-fold superfamily can also interact with proteins that do not belong to the superfamily. Fas receptor and FADD, containing a DD [7, 8], and FLIP (FLICE inhibitory protein), containing a DED [9], have been identified as calmodulin (CaM) target proteins.

CaM is a key calcium sensor protein involved in eukaryotic cells in a variety of cellular processes including apoptosis, cell cycle, inflammation and immune response [10]. CaM is composed of two globular domains, the N- and C-terminal lobes, linked by a flexible helix called the central linker. Each domain contains two helix-loop-helix EF-hand calcium-binding motifs [11, 12]. Upon calcium binding, CaM undergoes major conformational changes exposing hydrophobic target-binding surfaces in each of the globular domains [13–15]. These highly malleable surfaces allow binding and regulation of numerous, structurally diverse targets [16, 17]. CaM can also bind targets in the apo or partially saturated calcium forms. CaM contains nine highly conserved methionine residues. In mammalian CaM, four methionine residues are clustered in each of the globular domains at residues 36, 51, 71, and 72 in the N-terminal domain and at residues 109, 124, 144, and 145 in the C-terminal domain. A ninth methionine is located in the linker region at position 76. Due to their side-chain flexibility and hydrophobicity, methionine residues play important functions in Ca^2+^-bound CaM, stabilizing the open conformation and providing a target-binding interface [18]. The importance of methionine residues of CaM is also supported by their evolutionary conservation. For example, in *Tetrahymena* and *Dicytostelium*, all nine methionine residues have been preserved. In *Saccharomyces cerevisiae*, three methionine residues are conserved (Met 36, Met 72 and Met 124) and other six have been replaced by leucine [19]. Methionine residues in Ca^2+^-CaM are surface exposed and thus susceptible to oxidation [20]. The efficacy of several targets recognition and regulation by CaM is modified by methionine oxidation [21–36].

We have previously identified CaM as a FADD interacting protein and two binding sites in the C-terminal DD have been characterized [8]. FADD is a key adaptor protein transmitting apoptotic signals mediated by death receptors. It has also been implicated in multiple non-apoptotic functions including autophagic and necroptotic cell death, NF-kB activation, innate immunity, proliferation and cell cycle progression [37–39]. FADD and TRADD interact with each other and share important structural, functional features and subcellular localization. In particular, they have a C-terminal DD, are mediators of apoptotic and survival signals and are both involved in innate immunity pathways [40]. Here we used a database search and binding experiments, using recombinant proteins and cell lysates of hematopoietic and non-hematopoietic cell lines, to identify a putative CaM binding site in the α-helices 1–2 of TRADD.DD and to show that point mutations in α-helix 2 strongly impair TRADD-CaM interaction. Further, CaM oxidation and site-specific mutagenesis were used to show that both the N- and C-terminal lobes of CaM mediate CaM-FADD and CaM-TRADD interactions. Using extensively (hydrogen peroxide, H_2_O_2_ treatment) and partially (treatment of oxidized CaM with methionine sulfoxide reductases) oxidized CaM and site-directed Met-to-Leu CaM mutants for *in vitro* binding assays we demonstrated that: i) oxidation of all methionine residues decreases the affinity of CaM for both FADD and TRADD to undetectable levels; ii) methionine residues in both the N- and C-terminal lobes of CaM are involved in the interaction of CaM with FADD and TRADD; iii) treatments with both methionine sulfoxide reductases, MsrA and MsrB2, that completely repair oxidized CaM, restore the interaction of CaM with both FADD and TRADD.

## Material and Methods

### Cells, Antibodies, and Reagents

Human cell lines, HuT78 T cell lymphoma (ATCC) and U937 monocytic/macrophage (ATCC), were cultured in RPMI 1640 medium (BioWhittaker, Lonza, USA). Epithelial cells, HelaS3 and human embryonic kidney (Hek) 293T, were cultured in Dulbecco’s modified Eagle’s medium, 4.5 g/L glucose. Tissue culture media were supplemented with 10 mM Hepes pH 6.98–7.30, 1 mM L-glutamine, 100 U/ml penicillin/streptomycin (BioWhittaker) and heat inactivated 5% (HelaS3, Hek 293T) or 10% (all other cell lines) fetal bovine serum. All cells were cultured at 37°C in a 5% CO_2_ humidified incubator. Calmodulin sepharose 4B, protein G sepharose fastflow, protein A sepharose CL-4B and glutathione S-transferase (GST) sepharose 4B were from GE Healthcare Europe; EZview red and anti-Flag M2 affinity gel were from Sigma-Aldrich, Ni-NTA resin from Qiagen, Italy. Primary antibodies used were: GST goat polyclonal antibody (GE Healthcare Europe); FADD mouse IgG1 clone A66-2 (Becton Dickinson BD Pharmingen) and mouse Ig1 clone 1 (BD Transduction Laboratories); calmodulin mouse IgG1 (Upstate Biotechnology, Inc—UBI) and CaM I rabbit polyclonal (Santa Cruz Biotechnology, Inc.); TRADD mouse IgG2a (UBI); Flag and Flag-peroxidase M2 mouse IgG1 (Sigma-Aldrich); HA and HA-horseradish peroxidase (HRP) conjugated clone 12CA5 mouse IgG2b (Roche Applied Science). Sheep anti-mouse and anti-rabbit immunoglobulins HRP-conjugated were purchased from GE Healthcare Europe. CaM recombinant protein was from UBI, protease and phosphatase inhibitors were obtained from Roche Applied Science and Sigma-Aldrich.

### *E. coli* and mammalian expression vectors

pGEX-FADD and pEF-HA-FADD plasmids have been previously described [8]. TRADD full length open reading frame (ORF) and TRADD deletion mutants were produced by PCR using as template the IMAGE clone ID 3689007 and PCR fragments were cloned in pGEX expression vector (GE Healthcare, Europe). The QuickChange site-directed mutagenesis kit (Agilent Technologies, Inc Santa Clara, CA, USA) was used to generate point mutations in TRADD. Wild-type and TRADD mutants coding sequences were subcloned in pCMV-Tag2B expression vector 3’ to Flag (Agilent Technologies, Inc). The pET-15b-CaM plasmid [41] was kindly provided by Dr. M. Ikura (University Health Network, Ontario, Canada). The ORF of human methionine sulfoxide reductase, MsrA and murine MsrB2 were produced by PCR, using as template the Image clones 6504055 and 5150285, and cloned in the pQE-9 expression vector (Qiagen, Italy) 3’ to the 6xHis-tag coding sequence.

### Expression and purification of recombinant GST and His proteins

Recombinant GST- and His-tagged proteins were expressed and produced in BL21 (DE3) *E. coli* strain. For GST fusion proteins, *E. coli* transformed colonies were grown in 2XYT medium broth at 37°C until the OD_600_ reached 0.6–0.8, then 0.1 mM isopropyl-1-thio-{beta}-D-galactopyranoside (IPTG) was added and cells were grown for 3 h at 30°C or 37°C. Bacteria were lysed according to manufacturer’s directions. His-CaM protein was purified as previously described [8]. Human His-MsrA and murine His-MsrB2 enzymes were expressed and produced in *E. coli* cells, transformed colonies were grown in LB medium broth at 37°C to 0.8 OD_600_. Then, protein expression was triggered by addition of 1 mM IPTG. After 4 h of additional growth at 30°C, the bacteria were pelleted and resuspended in 20 mM Tris-HCl, pH 8.0, 0.7 M NaCl (buffer A) containing 20 mM imidazole, and disrupted by sonication. After centrifugation, the supernatants were purified with a Ni-NTA resin equilibrated in buffer A containing 20 mM imidazole, pH 8.0. After washing with buffer A containing 60 mM imidazole, the His-tagged proteins were eluted with buffer A containing 1 M imidazole.

### Expression and purification of Met-to-Leu CaM mutants

Met-to-Leu CaM mutants, previously described [34], were produced by site directed mutagenesis of CaM. Briefly, mutants were constructed from the wild-type rat CaM cDNA by using primer-based site-directed mutagenesis. DNA sequence analysis confirmed the correct generation of each mutant. Leucine was substituted for: i) all methionine (9L); ii) all but M109 (8L 109M); iii) all but M124 (8L 124M); iv) all methionine residues in the N-terminal domain along with M76 of the linker region (M36-76L); v) selected methionine in the C-terminal domain (M124-145L); vi-viii) combination of N- and C-terminal methionine residues (M36-76L; M51-72L; M36/M76-M145L) (Table 1). CaM mutants were expressed in *E. coli* BL21 (DE3) pLys5 strain, purified via phenyl-sepharose chromatography and dialyzed overnight at 4°C against 2 mM HEPES (pH 7.0) as previously described [42]. Protein concentration was determined by colorimetric assay using wild-type CaM as protein standard. CaM concentration was determined by the published molar extinction coefficient ε_277 nm–302 nm_ = 3,029 M^−1^ cm^−1^ [43]. Purified CaM mutants (0.5 μg) were loaded on 12 or 15% SDS-PAGE using Laemmli reducing sample buffer containing 1 mM EGTA. A single protein band on coomassie blue-stained gels (∼20 kDa) confirmed samples purity.

**Table 1:** Met-to-Leu CaM mutants.

**CaM mutants**		**CaM domains: Met and Leu positions**								
**Code**	**Description**	**N-terminal**				**Linker**	**C-terminal**			
		36	51	71	72	76	109	124	144	145
1	wild type	M	M	M	M	M	M	M	M	M
2	9L	L	L	L	L	L	L	L	L	L
3	8L 109M	L	L	L	L	L	M	L	L	L
4	8L 124M	L	L	L	L	L	L	M	L	L
5	M36–76L	L	L	L	L	L	M	M	M	M
6	M124–145L	M	M	M	M	M	M	L	L	L
7	M51–72L	M	L	L	L	M	M	M	M	M
8	M36/76–145L	L	M	M	M	L	L	L	L	L

Methionine residues located in the N-teminal (Met 36, 51, 71, 72), C-terminal (Met 109, 124, 144, 145) and linker region (Met 76) of CaM are listed. Mutant description reflects the relative position of Met-to-Leu substitutions as schematically indicated; 9L all nine methionine mutated, 8L 109M and 8L 124M eight methionine mutated with the exception respectively of residues 109M or 124M.

### *In vitro* oxidation of CaM methionines

CaM was oxidized essentially as previously described [26]. Briefly, 60 μM of purified CaM in 50 mM MES, pH 5.5, 1 mM MgCl_2_, 100 mM KCl, was incubated for 23 h with 50 mM H_2_O_2_ at room temperature. To stop the oxidation reaction, sample was dialyzed at 4°C against 10 mM sodium phosphate, pH 7.0, 100 mM KCl.

### *In vitro* reduction of CaM methionines by MsrA or/and MsrB2

Oxidized CaM (15 μM) in 10 mM sodium phosphate, pH 7.0, 100 mM KCl was incubated with 5 μM MsrA and/or 5 μM MsrB2 and 15 mM DTT (used as an electron donor, reducing agent) for 30 min at 37°C. Oxidation state and reduction efficiency of CaM was verified by denaturing 15% SDS-PAGE stained with coomassie blue. Samples were loaded in Laemmli sample buffer containing 2 mM Ca^2+^.

### CaM and GST pull-down assays

CaM binding assays were performed as previously described [8]. Briefly, *E. coli* GST-proteins or ^35^S-Met-labeled proteins, produced with a coupled transcription/translation kit (TnT Coupled Reticulocyte Lysate System; Promega, Italy) according to the manufacturer’s instructions, were diluted in binding buffer (50 mM Tris-HCl pH 7.6, 120 mM NaCl, 1% Brij 98) and incubated with calmodulin-sepharose 4B or control sepharose for 2 h at 4°C. After five washes, interacting proteins were eluted in 1 M NaCl, 2 mM EGTA and beads were boiled in reducing Laemmli sample buffer. Proteins were separated by SDS-PAGE and gels were either coomassie stained and exposed to phosphorimager or blotted to Hybond C nitrocellulose membranes (GE Healthcare, Europe) for western blot analysis. In GST pull-down assays with recombinant His-CaM, oxidized His-CaM (CaM_ox_), oxidized His-CaM repaired by treatment with MsrA (CaM_MsrA_) or MsrB2 (CaM_MsrB2_) or with both MsrA and MsrB2 (CaM_R_), no-tagged wild-type CaM and CaM Met-to-Leu mutants, the binding buffer was 10 mM potassium phosphate pH 7.0, 100 mM KCl and 1% Brij. In pull-down assays, 5 μg of CaM or GST proteins bound to beads were incubated with soluble GST or CaM proteins. To analyze pull-down assays, 1/20 of input (I) and 1/10 of eluates (E) were loaded on 12 or 15% (w/v) SDS-PAGE, if not otherwise specified. TRADD pull-down assays with ^35^S-Met-labeled FADD protein was essentially performed as previously described [44].

### CaM overlay experiments

Blot overlay assays with *Xenopus laevis* biotinylated His-CaM protein were performed as previously described [8].

### Cell transfection and immunoprecipitation assays

For TRADD-FADD *in vivo* interactions, Hek 293T cells were co-transfected with pCMV-Tag2B-TRADD and pEF-HA-FADD plasmids by the calcium phosphate method. Cells were harvested and lysed for 30 min on ice in lysis buffer (10 mM Tris-Cl, pH 7.4, 150 mM NaCl, 1 mM EDTA, 1 mM EGTA, 1% Triton X-100, 0.5% Nonidet P-40, protease and phophatase inhibitors) as previously described [44]. For the co-immunoprecipitation (IP) of TRADD mutants and FADD, 500 μg of cell extracts were incubated with Flag-resin at 4°C washed and processed following manufacture’s protocol. Total lysates and co-IP fractions were separated by 12% SDS-PAGE, blotted and probed with HA-HRP or Flag-HRP conjugated antibodies.

## Results and Discussion

### Identification of a calcium-dependent CaM binding site in TRADD

**Figure 1:**
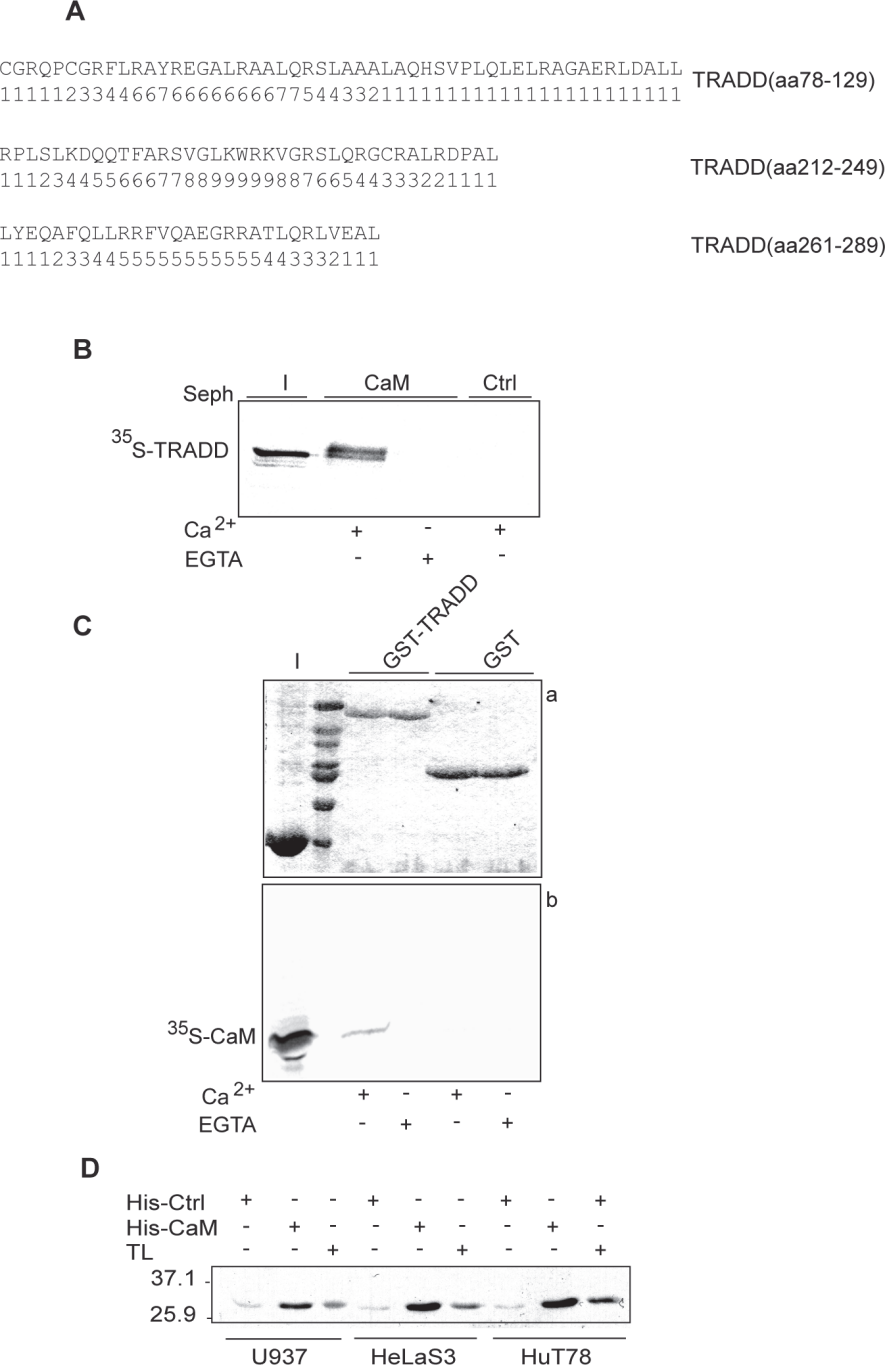
Ca^2+^-dependent binding of CaM to TRADD. **A**: CaM target database analysis. Amino acid sequences of the predicted CaM binding sites in human TRADD are shown along with the corresponding probability scores. **B**: autoradiography of CaM pull-down assay. (I) shows input of ^35^S-TRADD (∼ 33 kDa) incubated with CaM sepharose (CaM) or control sepharose (Ctrl) beads in Ca^2+^ or EGTA binding buffer. **C**: autoradiography of pull-down assay of ^35^S-CaM with GST or GST-TRADD. Panel a) shows a 12% SDS-PAGE stained with coomassie and panel b) the corresponding autoradiogram for ^35^S-CaM. **D**: western blot of His-CaM pull-down assays. His-CaM or His-Ctrl (control, cyclophilin) bound to Ni-NTA agarose beads were incubated with cell lysates, as indicated. TL indicates total cell lysates. Positions of the molecular weight standards are indicated. The data shown are representative of at least three independent experiments.

The database of Dr. Ikura’s laboratory (http://calcium.uhnres.utoronto.ca/ctdb) was used to search for putative CaM binding sites in TRADD [16]. Computational tools assign scores to putative binding sites within a protein sequence based on α-helical tendency, hydropathy, residue weight, net charge, hydrophobic residue content and occurrence of specific residues. Scores assigned to a twenty residues window are normalized for the entire sequence. In TRADD, three putative CaM binding sites are predicted: one located in the N-terminal domain (aa 78–129) and two in the C-terminal DD (aa 212–249; aa 261–289) (Fig. 1A). The most likely binding site is highlighted by a series of 9s [16].

To demonstrate a direct binding between TRADD and CaM, to investigate whether the interaction was calcium-dependent and to identify CaM binding sites in TRADD, we performed binding assays with: i) CaM-sepharose and soluble GST-TRADD fusion proteins or ^35^S-Met-labeled TRADD protein; ii) sepharose bound GST-TRADD and ^35^S-Met-labeled CaM protein. Binding assays, SDS-PAGE and autoradiography analyses show that ^35^S-Met-TRADD specifically binds to CaM-sepharose in a calcium-dependent fashion (Fig. 1B). Intriguingly, TRADD protein upon binding to CaM-sepharose migrates as a doublet in SDS-PAGE (Fig. 1B lane 2), suggesting that post-translation modifications or SDS-resistant conformational changes are induced in TRADD upon CaM-binding. In reciprocal experiments, GST-TRADD beads pull-down ^35^S-Met-labeled CaM in a calcium specific manner (Fig. 1C). Importantly, binding of TRADD to CaM was confirmed using endogenous human TRADD protein. Specifically, TRADD in cellular extracts of hematopoietic (HuT78), epithelial (HeLaS3) and monocytic/macrophagic (U937) cell lines are pull-down specifically with His-CaM nickel beads (Fig. 1D).

### Identification and characterization of a calcium-dependent CaM binding site in TRADD.DD

**Figure 2:**
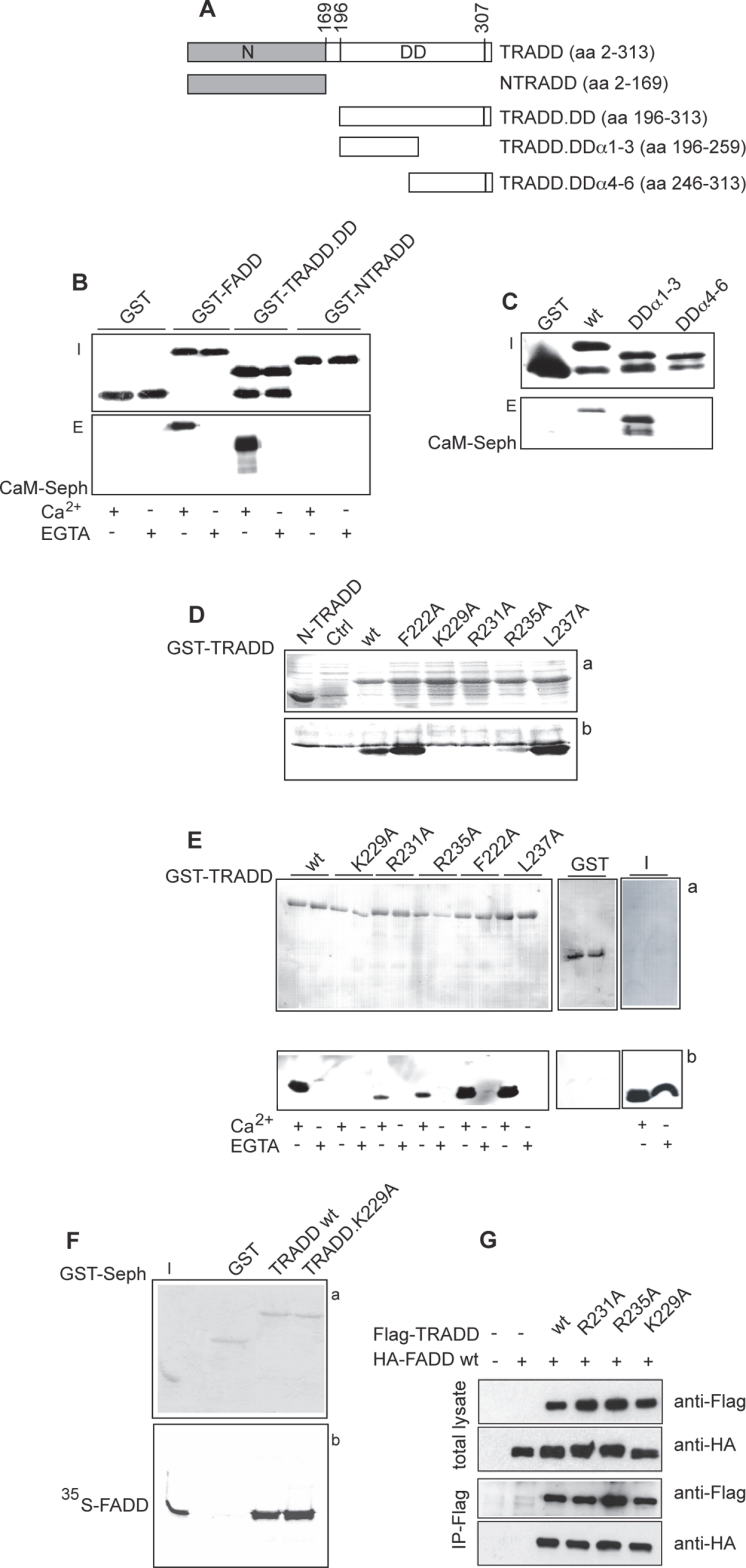
Characterization of a CaM binding site in TRADD.DD. **A**: schematic representation of the GST-TRADD mutants. The N-terminal domain (N) and the Death Domain (DD) of human TRADD are indicated. **B** and **C**: western blot with GST specific antibody of GST-TRADD mutants (top panels, I stands for inputs) and CaM pull-down assays (bottom panels, E stands for eluates). The GST fusion proteins indicated were pull-down with CaM-sepharose beads in binding buffer with 2 mM Ca^2+^ (**B, C**) or EGTA (**B**). **D**: CaM blot overlay assay. *E coli* BL21 lysates expressing N-TRADD (lane 1) or untransformed (Ctrl) (lane 2) were used as negative controls. Top panel a) shows the ponceau stained filter and bottom panel b) the western blot probed with biotin-conjugated His-CaM. **E**: GST-TRADD pull-down assays. The GST-TRADD proteins indicated, bound to glutathione-sepharose beads, were incubated with His-CaM in binding buffer with 2 mM Ca^2+^ or EGTA. Top panel a) shows the ponceau stained filter and bottom panel b) the western blot probed with CaM specific antibody. I indicates the input of His-CaM. **F**: GST-TRADD pull-down assays. Bound proteins were analyzed by 12% SDS-PAGE and autoradiography. Panel a) shows the coomassie stained gel where I indicates the input of ^35^S-FADD. Panel b) shows the corresponding autoradiogram. **G**: Immunoprecipitation (IP) assay. Total lysates of Hek 293T cells expressing HA-FADD and Flag-TRADD proteins were probed with Flag or HA monoclonal antibodies. Cell lysates were IP with Flag-resin, as described in material and methods. The data shown are representative of at least three independent experiments.

To further characterize the CaM binding sites, a series of GST-TRADD deletion mutants containing the: N-terminal domain (N-TRADD), C-terminal DD (TRADD.DD), α-helices 1–3 (TRADD.DD α1–3) or 4–6 (TRADD.DD α4–6) of TRADD.DD (Fig. 2A) were incubated with CaM-sepharose and bound proteins analyzed by western blot. The slower migrating bands in western blots (inputs and eluates) correspond to the expected molecular weight of full-length recombinant GST fusion proteins (GST ∼25kDa; GST-FADD ∼48kDa, GST-NTRADD ∼44kDa, GST-TRADD.DD ∼38kDa, GST-TRADD.DD**α**1–3 ∼32kDa, and GST-TRADD.DD**α**4–6 ∼32kDa), while the fast migrating bands likely correspond to degradation products. In Fig. 2B we show that CaM-sepharose binds TRADD.DD in a calcium-dependent fashion, but not N-TRADD; in the assays, GST-FADD and GST were, respectively, used as positive and negative controls. Thus, in our experimental conditions, no interaction of N-TRADD with CaM was detected even if a putative binding site, with a low score, was predicted by database search in this domain (Fig. 1A).

The structure of TRADD.DD consists of a canonical anti-parallel six helix bundle, characteristic of the DD superfamily, where α-helices-1 (L216-S225), -4 (L261-E276) and α-helices-2 (K229-G240) and -5 (L282-E291) lie on opposite side of the protein and α-helix-3 (A248-R258) and -6 (T295-L301) are located, respectively, at the bottom and on top of the bundle [45]. The DD putative CaM binding sites should be located respectively in the α-helices 1–2 with connecting loops (aa 212–249) and in the α-helices 4–5 with connecting loops (aa 261–289). CaM sepharose pull-down assays with the two truncated mutants, containing α-helices 1–3 or 4–6 of TRADD.DD (Fig. 2A), demonstrated that only TRADD.DD α1–3 binds in a Ca^2+^-dependent fashion to CaM (Fig. 2C). Therefore, the predicted putative binding site in α-helices 4–5 of TRADD.DD, with a low prediction score (Fig. 1A), was not confirmed.

Ca^2+^-dependent CaM binding motifs have been classified by the spacing between hydrophobic anchor residues (http://calcium.uhnres.utoronto.ca/) given the lack of a well-defined CaM binding consensus sequence [16]. The CaM binding site predicted in αhelices 1–3 of TRADD.DD (aa 212–249) (Fig. 1A) contains a putative 1–16 motif with 5 basic residues and a net charge of +5 (aa 222–237 FarsvglkwrkvgrsL) and an overlapping 1–10 motif with 4 basic residues and a net charge of +4 (aa 228–237 LkwrkvgrsL).

### Identification of TRADD mutations that interfere with TRADD-CaM interaction

The CaM binding domain of target proteins is usually a short peptide with propensity to form a α-helix that is hydrophobic-basic in nature [46]. In many classical Ca^2+^-CaM peptide complexes, the peptide is anchored through interaction of hydrophobic residues to hydrophobic CaM pockets whereas basic residues mediate electrostatic contacts with the highly acidic surface of CaM. Unclassified CaM binding sites have also been described [46]. For mutagenesis studies, we selected five residues within the predicted motifs in α-helices 1–3 of TRADD.DD: two hydrophobic (F222 and L237) and three basic (K229, R231 and R235). A series of GST-TRADD mutants containing single substitutions of alanine were produced and screened by blot overlay with biotin-labeled His-CaM. As shown in Fig. 2D, F222A and L237A TRADD mutants bind Ca^2+^-CaM in a manner that is essentially comparable to wild-type TRADD (Fig. 2D). K229A, R231A and R235A TRADD mutants, instead, dramatically reduce (R235A), or ablate (K229A, R231A), Ca^2+^-CaM binding as compared to wild-type TRADD (Fig. 2D). No signal was detected when the blots were incubated with CaM protein in a buffer containing EGTA (data not shown). Pull-down assays confirmed that K229A, R231A and R235A mutations in TRADD impair CaM binding. In fact, TRADD.K229A mutant does not bind CaM-sepharose and TRADD.R231A and TRADD.R235A reproducibly show a weaker binding to CaM (Fig. 2E). It is unlikely that K229A, R231A and R235A mutations induce major structural modifications in TRADD since it has been previously reported that TRADD.R231A and TRADD.R235A mutants can bind both TNFR1 and FADD proteins [44] and TRADD.K229A binds ^35^S-Met-labeled-FADD as wild-type TRADD (Fig. 2F). To further characterize the effects of K229A, R231A and R235A mutations, we ectopically expressed, in Hek 293T cells, Flag-TRADD mutants along with HA-FADD wild-type protein. Transfection efficiency was similar for all TRADD mutants and, accordingly, similar protein levels were detected in total cell lysates by western blot (Fig. 2G). Co-immunoprecipitation data indicate that wild-type TRADD, as well as TRADD.R231A, TRADD.R235A and TRADD.K229A interact with FADD (Fig. 2G). These results demonstrate that specific mutations in TRADD that impair CaM interaction do not alter the interaction of TRADD with FADD.

Overall, these results support the presence of a CaM binding site in the α-helix 2 of TRADD.DD. Although we did not detect interaction of CaM with N-TRADD, we cannot exclude the possibility that N-TRADD in the full-length protein might contribute to CaM-TRADD interaction. The predicted CaM binding motifs (1–16, 1–10) in α-helices 1–3 of TRADD.DD were not supported by our mutagenesis analysis; in fact the hydrophobic anchor residues F227A and L237A did not alter CaM-TRADD binding. Further mutagenesis and structural studies are needed to characterize the CaM binding motif in the α-helix 2 of TRADD.DD.

Several members of the death-fold superfamily, containing a DD, have been identified as CaM target proteins including Fas (CD95) receptor [7], FADD [8] and TRADD (this report). The interaction of CaM with death-fold proteins likely requires significant conformational changes in both proteins. It is well known that binding of Ca^2+^ to CaM triggers major structural rearrangements in both N- and C-terminal lobes resulting in accessibility of hydrophobic residues that are essentially buried in apo calmodulin [13–15]. However, in order for the interaction to take place also CaM interaction binding motifs in TRADD.DD and FADD.DD must be exposed. Notably, some plasticity has been reported for members of the death fold family [47]. Characterization of a crystallographic structure of Fas.DD and FADD.DD complex, containing four Fas.DDs and four FADD.DDs assembled in a dimer of two Fas.DD:FADD.DD complex dimers, highlighted remarkable structural changes in Fas.DD when compared to the unbound Fas.DD [48]. In the complex Fas.DD showed a significant open transition providing binding sites for FADD.DD, otherwise unavailable in the unbound closed form. The structural changes observed in the crystallographic complex led to the description of a “conditional domain interaction” model in which, upon Fas receptor clustering the open and unstable form of Fas receptors can interact and stabilize each other, making the binding sites for FADD available [48]. Notably, a FADD.DD conformational change, even if not dramatic as the opening of Fas.DD, was also observed and suggested to be potentially involved in the amplification of the “off-on” switch [48]. Under different experimental conditions Fas.DD predominantly interacted with FADD.DD in a 5:5 complex without major alteration of Fas.DD structure [49, 50]. Deeper characterizations, under physiological conditions, of the structural changes of Fas.DD and FADD.DD in multimeric complexes upon Fas receptor activation as well as the contribution of other interacting proteins in such complexes are very challenging.

Remarkably, conformation and free-energy studies of the CaM-Fas.DD complex revealed that CaM binding to Fas.DD results in conformational changes of both Fas and CaM and stabilization of both structures [51]. Moreover, a combination of structural and biophysical studies showed that two CaM molecules bind to Fas.DD and that both the N- and C-terminal lobes of CaM are involved. These findings further support the view that Fas.DD unfolding is probably required for CaM binding [52].

Taken together all these literature data provide the basis to hypothesize that binding of CaM to FADD or TRADD should result in conformational changes of both CaM and its targets.

### *In vitro* oxidation of CaM methionines drastically reduces CaM affinity for FADD and TRADD

CaM interacts with both FADD [8] and TRADD (this report). Two putative binding sites were identified and characterized respectively in the α-helices 8–9 and 10–11 of FADD.DD [8] and one binding site in the α-helix 2 of TRADD.DD. To assess the role of CaM methionine residues in its interaction to FADD and TRADD we performed binding assays with native (CaM_N_), oxidized (CaM_ox_), partially (CaM_MsrA_ or CaM_MsrB2_) or totally repaired (CaM_R_) CaM protein. CaM contains no cysteine residues and, under acid conditions, oxidation can be specific for methionine residues [53]. The *in vitro* extensive oxidation protocol used here converts all nine methionine residues of CaM to MetO [26], since, although H_2_O_2_ can potentially oxidize a number of amino acids, the thioether group of methionine is not protonated at low pH and therefore it can be selectively oxidized [53]. Less extensive oxidation can be achieved by treating oxidized CaM with MsrA or MsrB2 that produce CaM samples containing multiple oxiforms.

**Figure 3:**
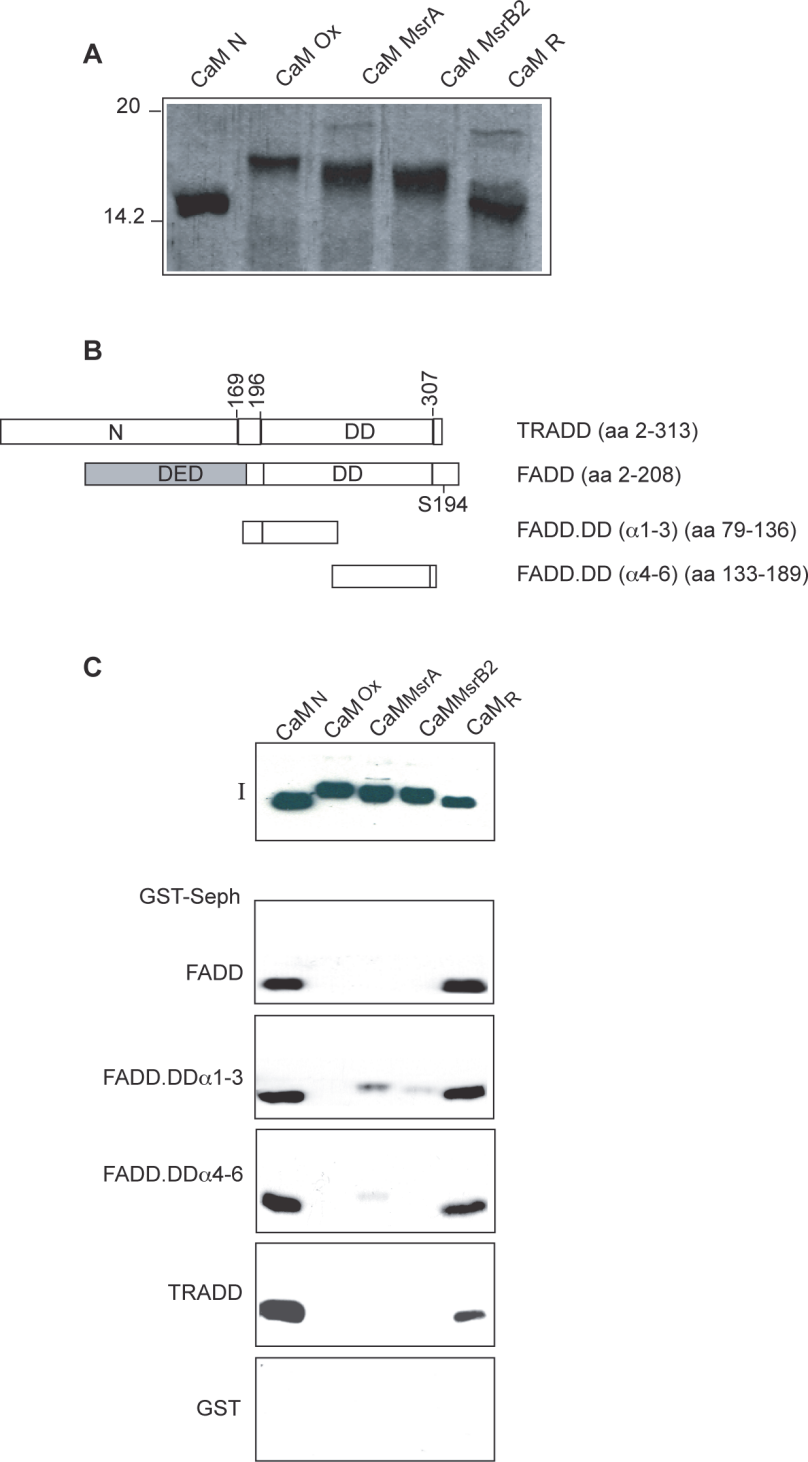
Methiones oxidation impairs CaM interaction with FADD and TRADD. **A**: CaM oxidation analysis. Coomassie blue stained 15% SDS-PAGE loaded with the indicated *Xenopus laevis* His-CaM proteins. **B**. Schematic representation of GST-FADD and GST-TRADD proteins used in pull-down assays. The DED, DD and the S194 phosphorylation site of human FADD are indicated. **C**: GST pull-down assays. The indicated GST proteins bound to gluathione-sepharose beads were incubated with recombinant His-CaM proteins, as indicated; 50% of the eluted proteins were subjected to SDS-PAGE (12%) and western blot analysis. I indicates the input of the CaM proteins used in binding assays. The data shown are representative of at least three independent experiments.

To study the effect of Msr on oxidized CaM, His-CaM_ox_ was incubated with either MsrA or MsrB2 using DTT as an electron donor in the reduction process. As shown in Fig. 3A, CaM_ox_ migrates slower than CaM_N_, most likely as a consequence of protein conformational alterations, as previously suggested [26], CaM_MsrA_ and CaM_MsrB2_ (“partially repaired CaM”) migrate in SDS-PAGE as several bands exhibiting different electrophoretic mobility, intermediate between that of His-CaM_N_ and His-CaM_ox_. These different species/bands, as previously suggested, most likely correspond to partially and heterogeneously reduced CaM molecules, having various combinations of oxidized and reduced methionines, with different conformations and/or affinities for calcium. Upon oxidation, methionine residues of CaM should be randomly converted to either the S or the R diastereoisomer of MetO and MsrA or MsrB2 could reduce only one of the two diastereoisomer [26]. Complete repair of methionine residues of CaM_ox_ was achieved upon incubation with both MsrA and MsrB2. The resulting CaM_R_, “fully repaired CaM”, migrates like CaM_N_ on SDS-PAGE suggesting that all nine MetO have been reduced (Fig. 3A). Next, pull-down assays were performed and the results clearly indicated that oxidation of methionine residues dramatically impairs CaM interaction with FADD, TRADD and FADD deletions mutants (schematically depicted in Fig. 3B) containing a single CaM binding site (Fig. 3C). Moreover, CaM_MsrA_ and CaM_MsrB2_ do not bind or bind weakly to FADD and TRADD, indicating that incomplete reduction of oxidized CaM by either MsrA or MsrB2 is insufficient to fully restore the interaction (Fig. 3C). Instead, CaM_R_ fully recovers the potency of native untreated CaM in GST-FADD and GST-TRADD binding (Fig. 3C). Similar results were obtained by blot overlay experiments, where biotinylated His-CaM_N_ and His-CaM_R_ decorated filters containing GST-FADD and GST-TRADD, while no signals were detected with His-CaM_ox_ (data not shown).

### N- and C-terminal methionine residues of CaM are critical for CaM binding to FADD and TRADD

**Figure 4:**
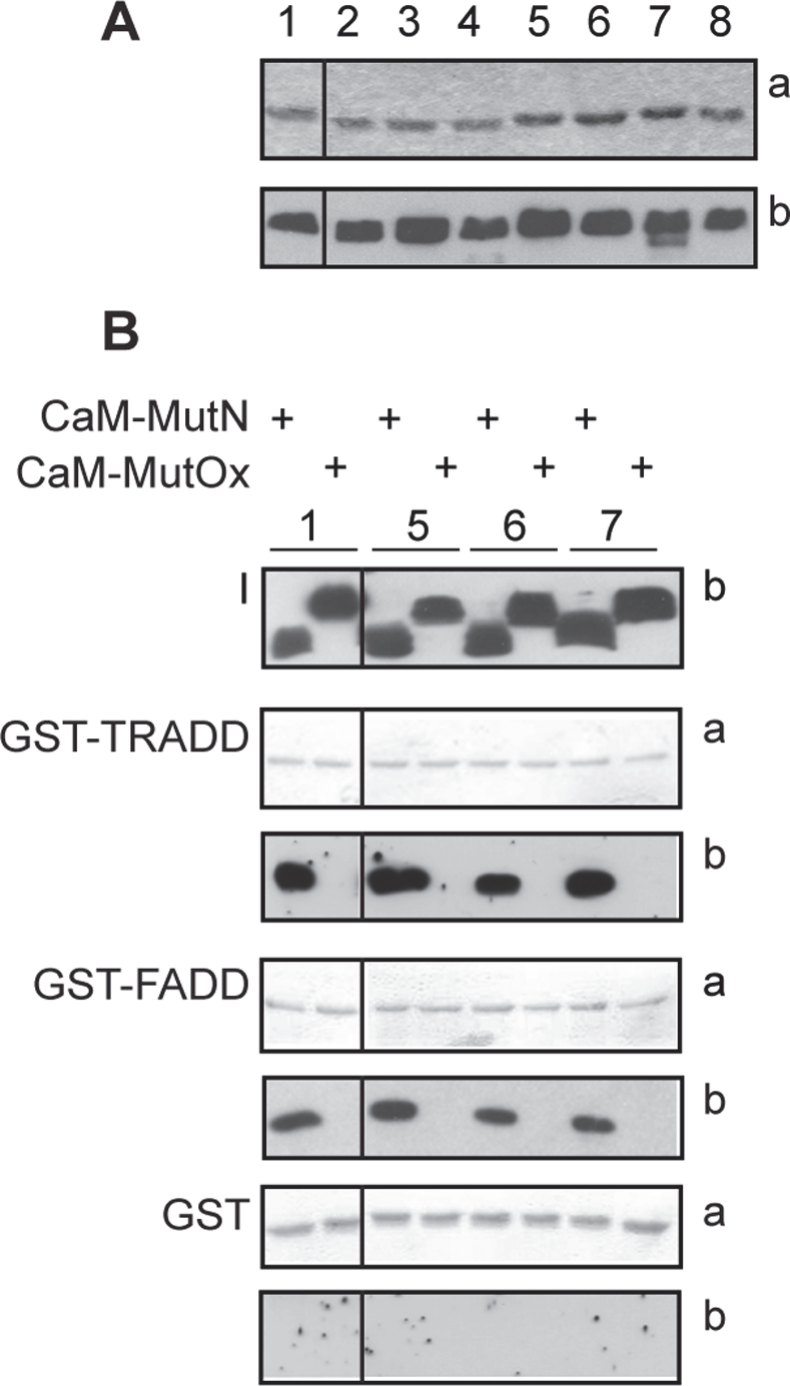
Effect of Met-to-Leu substitutions in CaM on FADD and TRADD binding. **A**: CaM mutants and SDS-PAGE analysis. Coomassie stained gel (a) and western blot analysis (b) of CaM mutants, identified by a numerical code detailed in Table 1. **B**: GST pull-down assays. Native (N) or oxidized (Ox) CaM mutants were incubated with the indicated GST proteins bound to glutathione-sepharose beads. 50% of the eluted proteins were analyzed by 12% SDS-PAGE and blotted to nitrocellulose stained with ponceau (a) and processed for western blot (b). I indicates the input of recombinant CaM proteins (250 ng). CaM mutants are identified by a numerical code as in Table 1. **A** and **B**: the black vertical lines in all panels indicate that non-adjacent lanes from the same gel or blots are shown. The data shown are representative of three independent experiments.

The role of the N- and C-terminal methionine-rich patches of CaM in FADD and TRADD binding was examined using a series of Met-to-Leu CaM mutants, schematically represented in Table 1 and previously described [34]. Mutation of Met-to-Leu is considered a conservative substitution based on data from evolution and the physico-chemical properties of the two amino acids. Throughout evolution, leucine is the most common substitution for methionine [54]. Leucine is slightly more hydrophobic than methionine, but both amino acids have similar volumes and propensity to form α-helices. Met-to-Leu substitutions are generally well tolerated by CaM. We previously used circular dichroism to assess the impact of Met-to-Leu mutations in CaM and found that up to 5 substitutions had no significant effect on the α-helical content of the molecule. Substitution of 6 or 9 methionine residues resulted in only a minor loss of α-helical content. Further, replacement of up to 6 methionines with leucines did not change CaM’s thermal stability [34]. Met-to-Leu CaM mutants were incubated with GST, GST-FADD and GST-TRADD and binding was detected by western blot with a CaM polyclonal antibody that recognizes all CaM mutants (Fig. 4). The binding assays showed that CaM mutants 9L, 8L 109M, 8L 124M, M36/76-145L do not bind FADD or TRADD (Table 2), suggesting that leucine is unable to substitute for methionine in mediating high affinity binding. All other CaM mutants bind specifically to both proteins (Fig. 4B, Table 2). It is likely that the CaM Met-to-Leu mutants that bind FADD and TRADD do so via the methionine residues remaining in the non-mutated either N- or C-terminal lobe. Specifically, the finding that the CaM mutant M36-76L binds to FADD and TRADD indicates that the C-terminal lobe mediates the interaction. The preserved competence of CaM mutant M124-145L allows to conclude that binding could also occur via the N-terminal lobe of CaM. Moreover, the inability of M36/76-145L CaM mutant to bind suggests that the remaining 3 N-terminal lobe methionine residues are insufficient to maintain binding. Based on these data, it was expected that oxidation of the remaining methionine residues in either the N- or C-terminal lobe of CaM should abolish CaM binding. Thus, wild-type and Met-to-Leu CaM mutants, that bind FADD and TRADD, were oxidized and tested for their ability to bind FADD and TRADD. SDS-PAGE and western blot analysis showed that all oxidized CaM mutants migrate slower than the corresponding native mutants and are recognized by a CaM polyclonal antibody (Fig. 4B). Native and CaM oxidized mutants were then used in binding assays with FADD and TRADD. As shown in Fig. 4B, oxidation abolishes CaM binding to TRADD and FADD, while, in the same experimental conditions, the native CaM mutants bind both proteins. The results with the oxidized CaM Met-to-Leu mutants further support the suggestion that methionine residues in both the N- and C-terminal lobes of CaM are critical for the interaction. Notably, both the N- and C-terminal lobes of CaM have been shown to be important for Fas.DD interaction [52]. Protein conformational plasticity of CaM has been proposed as a means of achieving functional diversity [17]. Plasticity of CaM at the level of individual amino acid side chains, in particular methionine residues, and in terms of orientation of the N- and C-terminal lobes is crucial for recognition and regulation of more than three hundred CaM targets [16, 17]. CaM contains nine methionines corresponding to 6% of the entire sequence, which is significantly higher than the average of known proteomes (1%). The relevance of methionines in interacting with target proteins has emerged from a number of structures of CaM in complex with its targets, where CaM can adopt largely different conformations [17]. Proteins that interact with a large number of partners play a central role in the organization of protein interaction networks [55]. Interestingly, CaM protein has been shown to undergo post-translational modifications including acetylation, trimethylation, carboxylmethylation, proteolytic cleavage, and phosphorylation and to be highly susceptible to methionine oxidation. Indeed, oxidized CaM has been isolated from nitric oxide synthase isoforms of aged animals [56, 57]. Overall, the literature data clearly indicate that methionine residues are important for CaM binding and that CaM functions can be modulated by the redox status of its methionine residues: oxidation could lower CaM affinity for calcium [33], could impair directly or indirectly targets recognition [21–32] and/or increase CaM susceptibility to degradation by the proteasome [34–36]. For example, oxidized CaM is unable to properly activate the plasma membrane Ca^2+^-ATPase [22–25], the *Bordetella pertussis* adenylate cyclase [26], the ryanodine receptor calcium channel RyR1 and RyR2 [27–28] and some protein kinases [30, 31].

**Table 2:** Binding assays of Met-to-Leu CaM mutants.

**Code**	**Description**	**GST**	**GST-FADD**	**GST-TRADD**
1	wild type	−	+	+
2	9L	−	y	−
3	8L 109M	−	−	−
4	8L 124M	−	−	−
5	M36–76L	−	+	+
6	M124–145L	−	+	+
7	M51–72L	−	+	+
8	M36/76–145L	−	−	−

Oxygen radicals and other reactive oxygen species (ROS) may lead to damage of nucleic acids, lipids and proteins [58], but can also modulate cell signaling, gene expression, cell death, cell cycle, proliferation and cell differentiation [59–62]. This dual function of ROS, due probably to differences in local concentrations, pulse duration and sub-cellular localization, is maintained through a delicate balance between production and removal of oxidants using both enzymatic and non-enzymatic processes. Cysteine and methionine amino acids are very sensitive to oxidation by ROS. The damage of most oxidized proteins is non-repairable, and has consequences on protein structure and function, although certain oxidation products of cysteine and methionine can be repaired [63, 64]. The major fate of unrepaired oxidized proteins is catabolism by proteosomal pathways to avoid their toxic accumulation within cells [65].

Our results suggest that *in vivo* oxidation of CaM could alter CaM binding to TRADD and FADD and in turn regulate FADD and TRADD functions.

## Conclusions

The majority of literature data in the last decade on death-domain proteins concern biochemical and functional studies on homotypic interactions [66]. The crystal structures of multimeric DD complexes, such as PIDDosome [67] Fas-FADD [48], MyDDosome [68], RIPoptosome [69], demonstrate that a single DD can engage in up to six interactions through three distinct and well-defined interaction types involving different helix/loop combinations in the interacting DDs. Little is known about the mechanisms regulating dynamic DD complex assembly in response to extracellular and intracellular changes including protein concentration, compartmentalization, protein folding and post-translational modifications. The interaction of DD proteins with CaM can modulate signaling pathways mediated or not by multimeric complexes [8–10]. Roles of TRADD-CaM interaction in death receptors signaling or death independent functions remain to be determined. Interestingly, TRADD mutants that impair CaM binding can still mediate interaction of TRADD with DD partners, such as TNFR1 and FADD, and thus could be instrumental to investigate the impact of CaM on TRADD-dependent complexes assembly.
